# Indocarbocyanine–Indodicarbocyanine (sCy3–sCy5) Absorptive Interactions in Conjugates and DNA Duplexes

**DOI:** 10.3390/molecules30010057

**Published:** 2024-12-27

**Authors:** Evgeny L. Gulyak, Vladimir A. Brylev, Mikhail Y. Zhitlov, Olga A. Komarova, Alexey V. Ustinov, Ksenia A. Sapozhnikova, Vera A. Alferova, Vladimir A. Korshun, Daniil A. Gvozdev

**Affiliations:** 1 Shemyakin-Ovchinnikov Institute of Bioorganic Chemistry, Miklukho-Maklaya 16/10, 117997 Moscow, Russia; evgeny.gulyak@gmail.com (E.L.G.); v.brylev@yandex.ru (V.A.B.); droplbox38@gmail.com (M.Y.Z.); okomarova802@gmail.com (O.A.K.); austinov@yandex.ru (A.V.U.); ksapozh@mail.ru (K.A.S.); alferovava@gmail.com (V.A.A.); 2 Department of Chemistry, Lomonosov Moscow State University, Leninskie Gory 1-3, 119991 Moscow, Russia; 3 Higher Chemical College of the Russian Academy of Sciences, D. Mendeleev University of Chemical Technology of Russia, Miusskaya Square 9, 125047 Moscow, Russia; 4 Department of Biology, Lomonosov Moscow State University, Leninskie Gory 1-12, 119234 Moscow, Russia

**Keywords:** sulfocyanine dyes, absorption spectra, dyes interaction, energy transfer, oligonucleotide duplex

## Abstract

Sulfonated indocyanines 3 and 5 (sCy3, sCy5) are widely used to label biomolecules. Their high molar absorption coefficients and lack of spectral overlap with biopolymers make them ideal as linker components for rapid assessment of bioconjugate stoichiometry. We recently found that the determination of the sCy3:sCy5 molar ratio in a conjugate from its optical absorption spectrum is not straightforward, as the sCy3:sCy5 absorbance ratio at the maxima tends to be larger than expected. In this work, we have investigated this phenomenon in detail by studying the spectral properties of a series of sCy3-sCy5 conjugates in which the dyes are separated by linkers of various lengths, including DNA duplexes. It was found that when sCy3 and sCy5 are located in close proximity, they consistently exhibit an “abnormal” absorbance ratio. However, when the two dyes are separated by long rigid DNA-based spacers, the absorbance ratio becomes consistent with their individual molar absorption coefficients. This phenomenon should be taken into account when assessing the molar ratio of the dyes by UV-Vis spectroscopy.

## 1. Introduction

**Figure 1:**
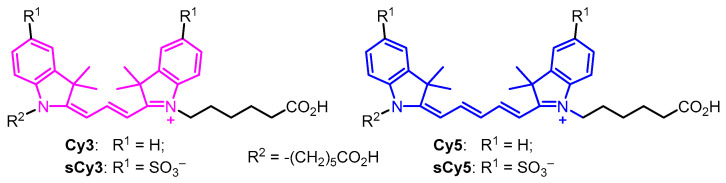
Structures of cyanine dyes (s)Cy3/5; the chromophore is highlighted by color.

Cyanine dyes are commonly used to label biomolecules [1,2,3,4,5], and a wide range of them are available to researchers. Among such labels, the simplest indocyanine dyes Cy3 and Cy5 (Figure 1) are highly valued for several reasons: (1) their main absorption and fluorescence bands are in the visible range and do not overlap with the absorption and fluorescence of main biomolecules classes; (2) they exhibit considerable molar absorption and rather high fluorescence quantum yields, resulting in appreciable fluorescence brightness in the 560–570 and 660–670 nm regions, respectively; Cy3 emits in the region of maximum sensitivity of the human eye; (3) Cy3 and Cy5 dyes are an effective FRET pair, which is used in the study of structural interactions in biomolecules (including single-molecule FRET [6,7,8,9,10]); (4) the dyes are rather photostable, possess excellent chemical stability, and are widely available as various functional/reactive derivatives suitable for coupling to biomolecules through a series of bioorthogonal reactions.

Water-soluble sulfonated derivatives sCy3 and sCy5 (Figure 1) have spectral properties very similar to those of the parent Cy3 and Cy5. When moving to sulfonated derivatives, the formal charge of the chromophore core changes from +1 to –1; however, in sulfocyanines, two ionic sulfo groups are exposed to the environment and are effectively solvated. Therefore, sulfonated cyanines are much more soluble in water, do not tend to aggregate, and interact much less with proteins, and especially nucleic acids, due to the electrostatic repulsion of negatively charged sulfonates and phosphates. In addition, of the two methyl groups in the 3-position of indole fragments of (s)Cy3/5 dyes prevent intercalation or placement of the dye molecule into the minor groove of DNA duplexes [11]. Thus, the sCy3/sCy5 pair has been used many times in various FRET experiments on nucleic acids [12,13,14,15,16,17].

A huge number of studies are devoted to various aspects of the use of (s)Cy3/5 dyes, and interesting results continue to appear. Thus, in recent years, the PIFE effect (earlier: protein-induced fluorescence enhancement; now: photoisomerization-related fluorescence enhancement) has been extensively studied, allowing one to obtain valuable structural information [18,19]. Very interesting findings were made on cyanine photobleaching/photoconversion [20,21,22,23,24].

The presence of two aromatic moieties in the indocarbocyanine molecule and the availability of various approaches to their functionalization naturally leads to the idea of using these dyes as colored/fluorescent linkers [25,26,27,28,29]. In particular, (s)Cy3/5 dyes are readily available as diacid derivatives (Figure 1, R^2^ = −(CH_2_)_5_CO_2_H). Recently, we used the absorption of sCy3 (ε_548_ 162,000 M^−1^cm^−1^) and sCy5 (ε_646_ 271,000 M^−1^cm^−1^) to directly assess the degree of modification (conjugate stoichiometry) of immunoglobulins upon fluorescent labeling [30] and to obtain antibody–drug conjugates (ADCs) [31]. At the same time, we noticed that, during a covalent connection with a linker containing both sCy3 and sCy5, the absorption ratio of sCy3 and sCy5 at their maxima is ~0.9, which is significantly higher than the expected value (ε_548_ sCy3 + ε_548_ sCy5)/ε_646_ sCy5 = (1.62 × 10^5^ + 1.20 × 10^4^) M^−1^cm^−1^/(2.71 × 10^5^) M^−1^cm^−1^ = 0.64). This observation led us to the need to make an empirical correction when assessing the degree of antibody modification.

**Figure 2:**
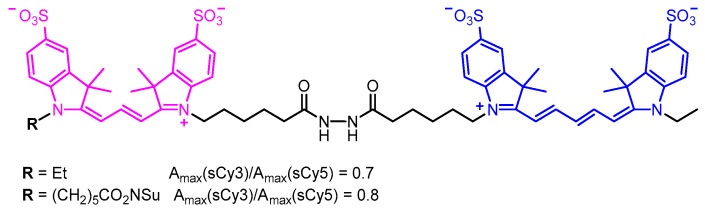
The structures of sCy3-sCy5 conjugates and their maximum absorbance ratios as reported by Conley et al. [38].

A number of examples of both covalent and non-covalent constructs containing (s)Cy3 and (s)Cy5 in close proximity have been reported [32,33,34,35,36,37,38,39,40,41]. However, to the best of our knowledge, only four such works contain information on the molar absorption coefficient ratio at Cy3 and Cy5 maxima. In the only publication concerning sulfonated dyes, Conley et al. [38] measured the absorbance ratios of 0.7 and 0.8 in water for two covalent sCy3-sCy5 conjugates (Figure 2), noting a slight perturbation in the relative oscillator strengths of the two dyes. It is worth mentioning that, in the absorption spectra extracted from HPLC traces of same conjugates (measured in a ~1:1 mixture of 0.1 M aqueous TEAA buffer and MeCN), the close-to-theoretical ratios of ~0.65 were observed.

Three other examples concern non-sulfonated Cy3-Cy5 conjugates. While a low-molecular-weight dimer reported by Jia et al. [41] exhibited a theoretically expected ratio of 0.72, the ratio in oligonucleotide-based constructs disclosed by Di Fiori et al. [39] and Lee et al. [40] varies from 0.37 to 0.86 depending on the relative spatial orientation on the dyes.

Overall, it is not immediately clear why the absorption spectrum of a covalent sCy3-sCy5 conjugate would exhibit any abnormality. Since the reference molar absorption values of the dyes are beyond doubt, one could assume that the reason underlying the phenomenon is the distance between the dyes: if it is short enough, some kind of interaction is realized, leading to a distortion of the absorption ratio at the maxima. We decided to study the interaction of chromophores sCy3 and sCy5 in more detail, placing them at certain distances from each other. To vary the distances between the dyes, we synthesized sCy3-sCy5 conjugates on linkers of various lengths, including using DNA duplexes to place the sCy3 and sCy5 molecules at a certain distance from each other. Here we report several such bichromophore conjugates, their spectral and photophysical studies, and practical implications for the use of the dyes as linkers for biomolecules.

## 2. Results and Discussion

### 2.1. Synthesis of Conjugates

**Figure 3:**
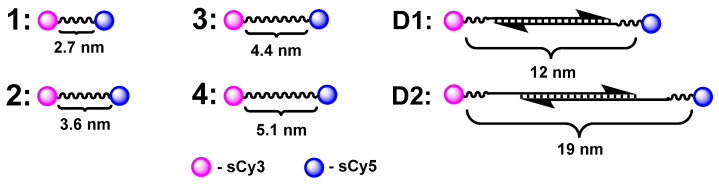
The sCy3–sCy5 conjugates used in this study: covalently linked (**1**–**4**) and based on oligonucleotide duplexes (**D1**, **D2**).

Four covalent (**1**–**4**) and two DNA oligonucleotide hybridization conjugates (**D1**, **D2**) were synthesized, varying the distance between the chromophores (Figure 3). The low molecular conjugates had flexible hydrophilic linkers, and the oligonucleotide constructs contained rigid (duplexes) and semi-rigid (oligo-T) elements.

**Figure 4:**
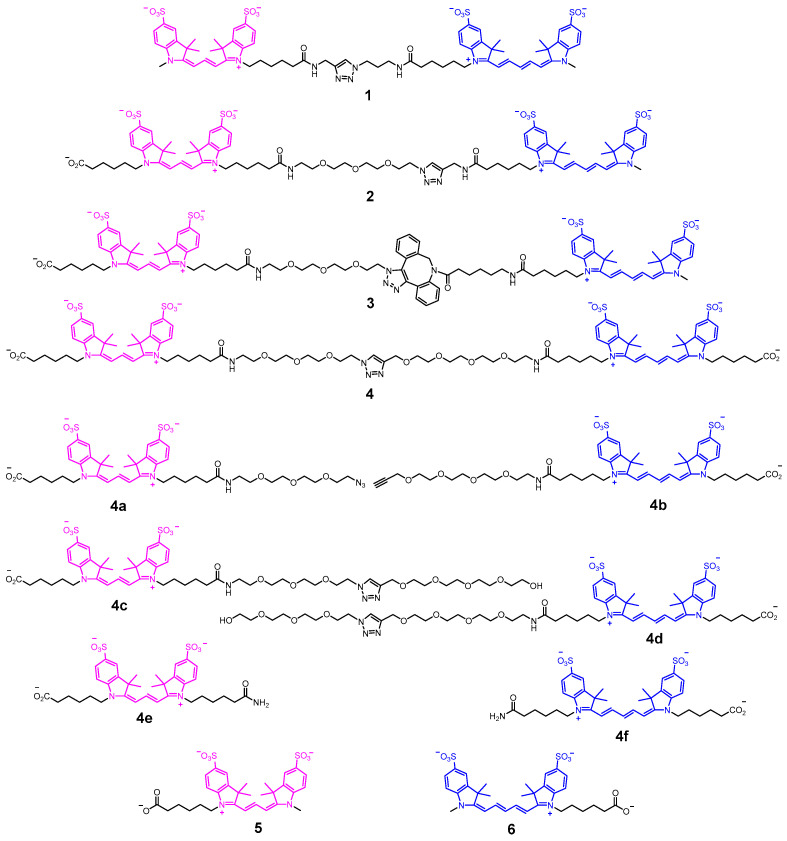
Structures of sCy3-sCy5 conjugates **1**–**4** and model compounds **4a**–**f** and **5**, **6**.

Low molecular weight conjugates **1**–**4** and model monomeric dye derivatives **4a**–**e** (Figure 4) were synthesized using CuAAC, SPAAC, and amine acylation as described previously [30]. Monomeric sCy3 and sCy5 derivatives **4a**–**f** and **5**, **6** contain variations of linker elements to take into account their influence on the spectral and photophysical properties of individual fluorophores.

**Figure 5:**
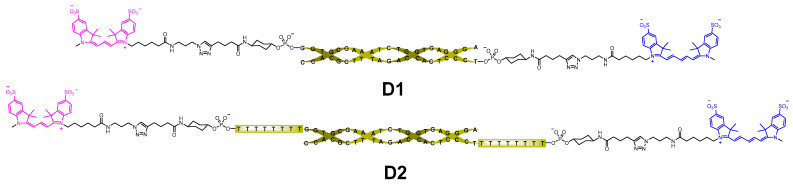
Structures of sCy3-sCy5 oligonucleotide conjugates **D1** and **D2**.

Oligonucleotide conjugates **D1** and **D2** were based on the 21-mer DNA duplex GGTCGCTTATCTGCACTCGGA/TCCGAGTGCAGATAAGCGACC. In conjugate **D2**, the duplex contained an additional extension of T_8_ dangling sequences (Figure 5). The starting 5′-alkynyl oligonucleotides were coupled with sCy3 and sCy5 azides using CuAAC [42,43,44] to yield the corresponding labeled oligonucleotides, which were then hybridized to yield target conjugates **D1** and **D2** according to a standard protocol [44] and purified using native PAGE (see Supporting Information, Table S1, Figures S1 and S2).

### 2.2. Free sCy3 and sCy5 Dyes Only Interact Weakly in Aqueous Solution

**Figure 6:**
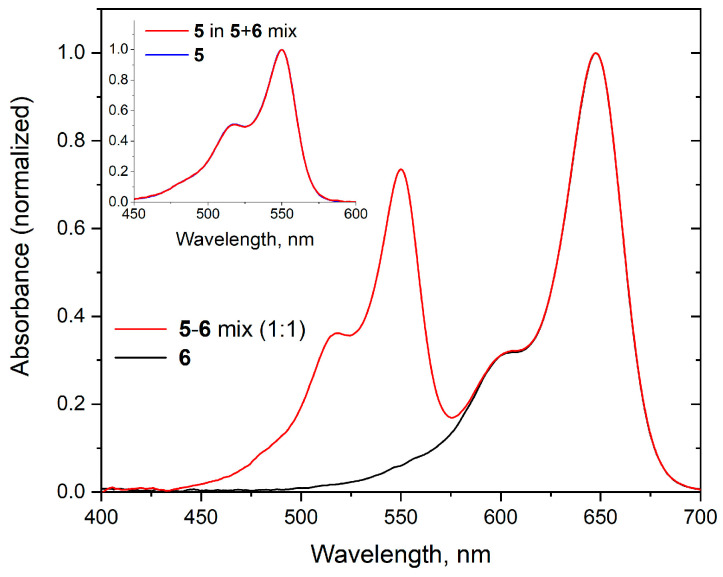
Absorption spectra of **6** and the equimolar mixture **5** + **6** in PBS. Inset: absorption spectra of **5** in PBS and **5** calculated by subtracting the spectrum of **6** from the **5** + **6** mixture spectrum.

In order to investigate whether the abnormal absorbance ratio is caused by covalent conjugation of the dyes, we examined the optical absorption of an equimolar mixture of individual sCy3 and sCy5 dyes. Solutions of dyes **5** and **6** and their 1:1 mixture **5**+**6** were diluted appropriately with PBS, and their absorption spectra were recorded (Figure 6). Absorption spectra of cyanine dyes contain two main subbands—0–0 major band and 0–1 minor (shoulder) vibronic band [45]. The maximum absorbance ratio (sCy3 0–0 band vs. sCy5 0–0 band) turned out to be slightly higher than expected (0.73 vs. the calculated value of 0.64). Thus, some interaction between dyes **5** and **6** was observed even at high dilutions (according to the Wigner–Seitz radius approximation, the average distance between sCy3 and sCy5 chromophores in the mixed solution is about 40 nm). The intensity ratios for 0–1/0–0 bands for individual sCy3 and sCy5 are 0.50 and 0.33, respectively, and remain the same in the mixture (Figure 6).

**Figure 7:**
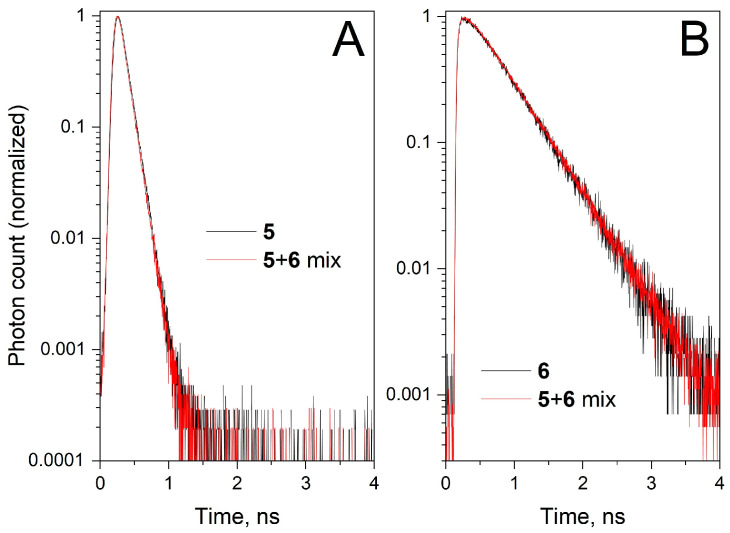
Fluorescence decay kinetics of **5** (**A**) and **6** (**B**) measured in PBS for individual solutions and an equimolar mixture. (**A**) sCy3 fluorescence, ex. 510 nm, em. 560 nm; (**B**) sCy5 fluorescence, ex. 640 nm, em. 680 nm.

At the same time, the shape of the absorption spectrum of individual **5** and **6** remained the same in the **5** + **6** mixture, indicating that there is no perturbation of the electronic density in the dyes in such an interaction (Figure 6). There was also no difference in the fluorescence decay curves of **5** and **6** in individual PBS solution and in the case of **5** + **6** mixture (Figure 7), indicating that no dynamic fluorescence quenching or FRET occurred in the **5** + **6** mixture. All these effects were the same in PBS and mQ water solutions of **5** and **6** (see Supporting Information, Figures S3 and S4). Thus, we observed only insignificant weak interactions for free cyanine dyes in aqueous solutions.

### 2.3. The Linker Affects the Absorption Spectrum and Fluorescence Lifetime of the Cyanine Dyes

In order to reveal the nature of the spectral changes of the cyanine dyes in the covalent conjugates, conjugate **4** was selected for detailed study. The sCy3:sCy5 0–0 band intensity ratio in conjugate **4** was found to be 1.29 and concentration-independent. In the range of optical densities from 0.05 to 1.0, **4** obeyed Beer’s law (the same was true for the individual dyes **5** and **6**). Absorption and fluorescence excitation spectra match in the case of **4**, **5**, and **6**, so no aggregates/dimers of cyanine dyes could be observed.

**Figure 8:**
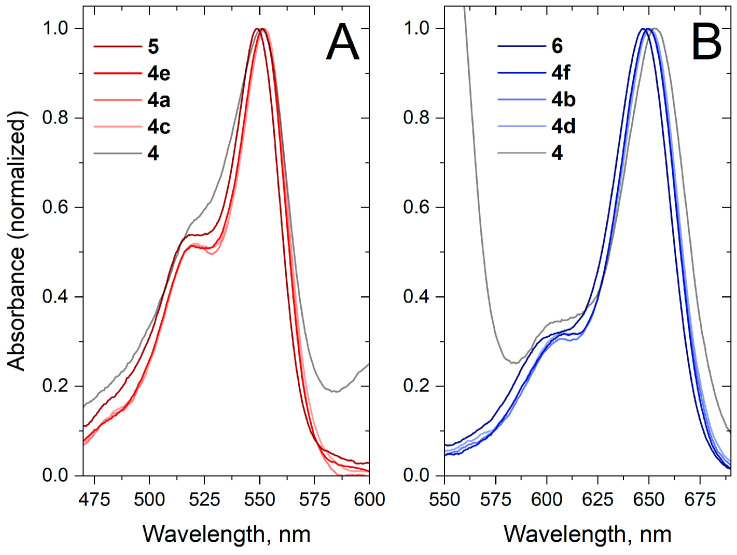
Normalized absorption spectra of (**A**) **4**, **4a**, **4c**, **4e**, **5** and (**B**) **4**, **4b**, **4d**, **4f**, **6** in 10 mM PBS.

**Figure 9:**
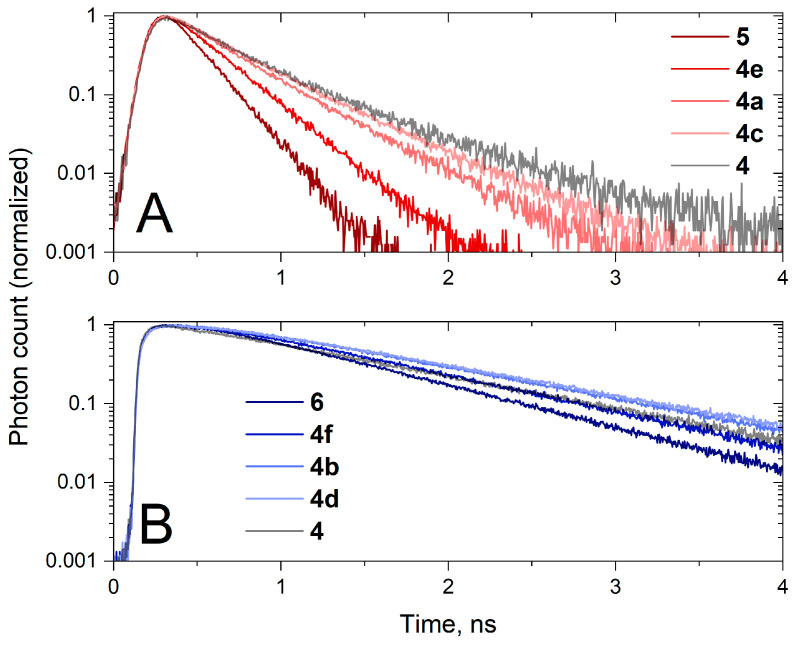
Fluorescence decay kinetics of an equimolar **4**, **4a**–**f**, **5**, and **6** measured in 10 mM PBS. (**A**) sCy3 fluorescence, ex. 510 nm, em. 560 nm; (**B**) sCy5 fluorescence, ex. 640 nm, em. 680 nm.

Next, we sought to confirm that the increase in the maximum absorbance ratio observed in **4** was caused by the conjugation of two dyes and not simply the linker. For that, we synthesized a series of controls **4a**–**f** and studied their spectral properties (Figure 8). While it was found that the linker does affect the spectral properties of the dyes, inducing a slight bathochromic shift, this was not sufficient to explain the anomalous absorption spectrum of **4**. The PEG-based linkers are capable of forming intramolecular hydrogen bonds, adopting coiled conformations. This effect alone can cause a slight perturbation of the electron density in the cyanine dye molecules, as seen in Figure 8, but, in the presence of the second dye in **4**, the perturbation is more pronounced. Fluorescence decay kinetics was also measured for dye-linker controls **4a**–**f** (Figure 9); it is clear that increasing the linker size leads to an increased **5**/**6** fluorescence lifetime, with more influence on sCy3.

Cyanine dyes are known to be capable of *cis*–*trans* isomerization, with the *cis* isomer barely fluorescent; the higher the probability of isomerization, the shorter the fluorescence lifetime of the dye [46]. Cy5 has greater rigidity than Cy3 due to a longer chain of conjugated bonds in the center of the molecule; Cy5 isomerizes with lower efficiency and therefore has a longer fluorescence lifetime [47]. The addition of a linker to the cyanine molecule increases the rigidity of the molecule, thereby causing a decrease in the probability of photoisomerization and an increase in fluorescence lifetime.

**Figure 10:**
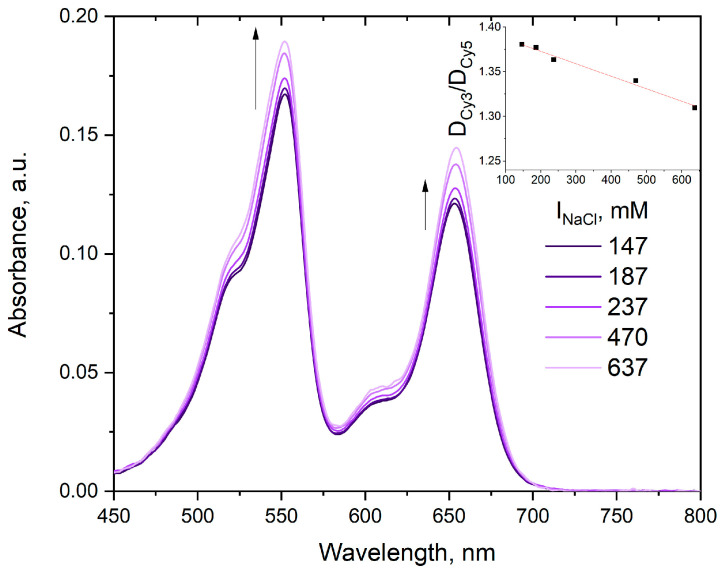
Absorption spectra of conjugate **4** at constant concentration in PBS in the presence of NaCl at various concentrations. Inset: absorbance ratios (sCy3 0–0 band vs. sCy5 0–0 band) at various NaCl concentrations.

**Figure 11:**
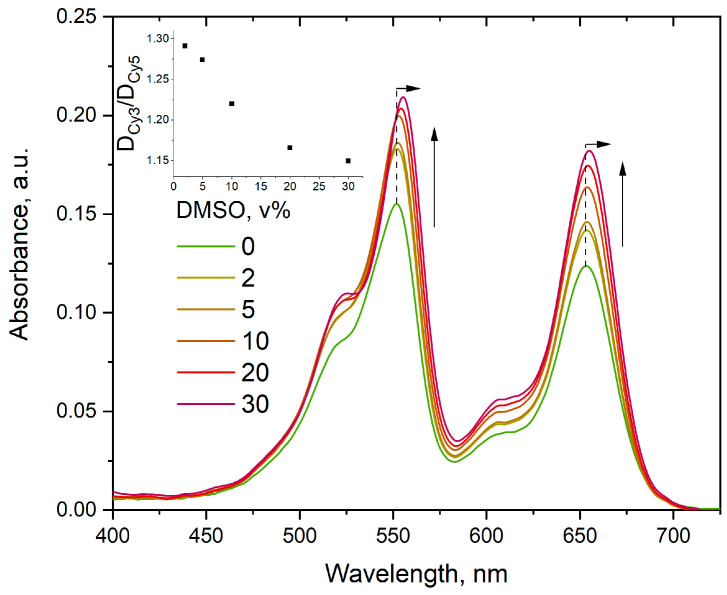
Absorption spectra of conjugate **4** at constant concentrations in deionized water in the presence of various DMSO concentrations. The arrows show change in spectral properties of the conjugate with increasing DMSO concentration. Inset: ratio of absorbances at maxima in the same sample as a function of DMSO concentration in solution.

The absorption spectrum of cyanine dyes connected by a linker depends on solvent properties. We found that an increase of ionic strength attenuates the sCy3/sCy5 maximum absorbance ratio in **4** (Figure 10). We additionally observed that higher ionic strength results in a slight bathochromic shift on the order of 1 nm and an increase in the molar absorption coefficients for both dyes in the conjugate. Curiously, this effect was only characteristic for the sCy3–sCy5 conjugate: individual dyes **5** and **6** react to the increase of ionic strength in a completely different manner, although a slight bathochromic shift is also observed (see Supporting Information, Figure S5). The same effect was observed for added DMSO for both conjugate **4** (Figure 11) and the individual dyes (see Supporting Information, Figure S6).

### 2.4. Comparison of the Spectral and Photophysical Properties for Various sCy3-sCy5 Conjugates

**Figure 12:**
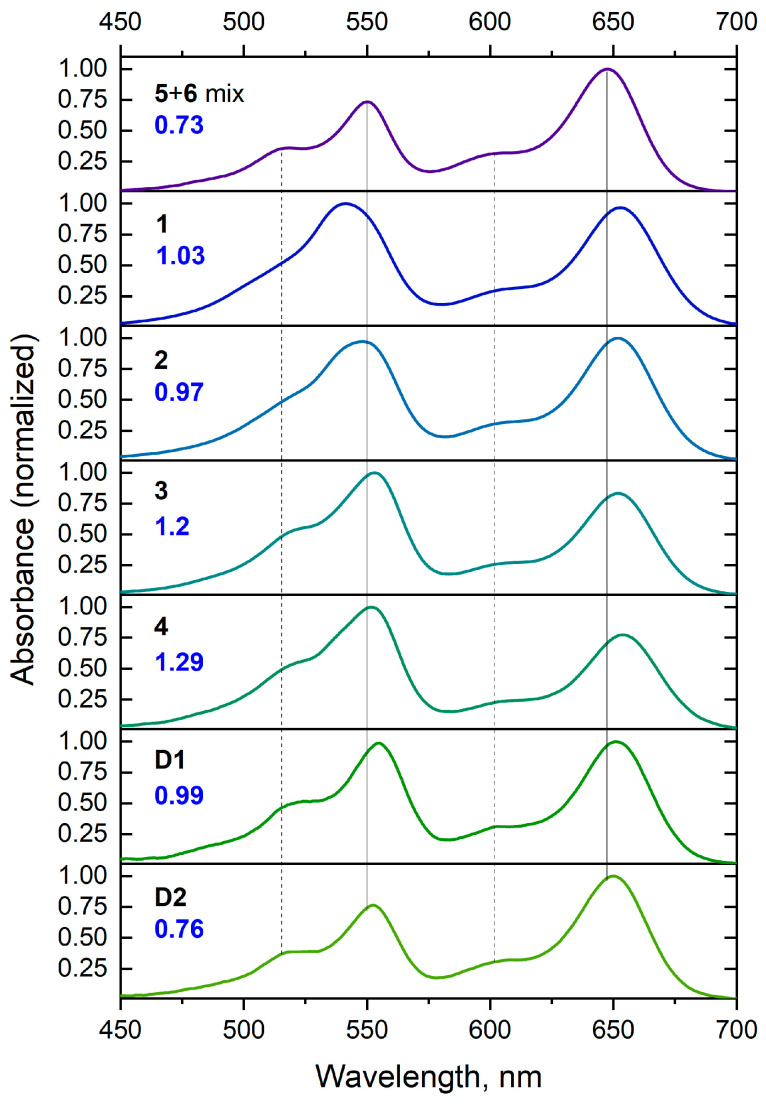
Absorption spectra of the **5** + **6** equimolar mixture, low molecular weight sCy3–sCy5 conjugates **1**–**4**, and oligonucleotide conjugates **D1** and **D2** in PBS. Blue numbers indicate the maximum sCy3/sCy5 absorbance ratio.

The absorption spectra of conjugates **1**–**4** and **D1**, **D2** were measured to examine their maximum absorbance ratios (Figure 12). Much higher intensity ratios of the sCy3 0–0 band to the sCy5 0–0 band were observed as compared to the values for the single dyes or the dye mixture; moreover, the maximum absorbance ratio increased with linker size. Large perturbation of the electronic density of sCy3 was observed with decreasing linker sizes. At the same time, sCy5 was less sensitive to such effects, probably due to a larger chain of conjugated bonds in the center of the molecule: the 0–1/0–0 band intensity ratios for sCy5 in conjugates **1**–**4** were identical (0.32), similar to the value for a single dye.

**Figure 13:**
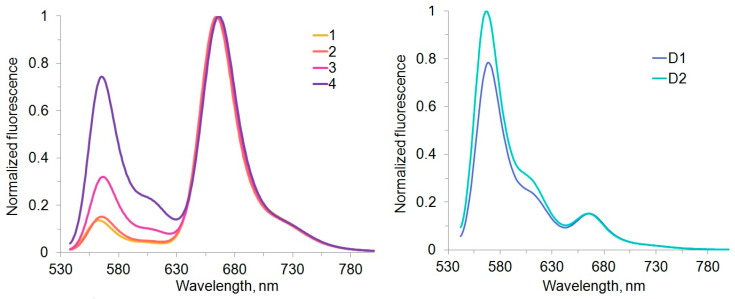
Normalized fluorescence spectra of conjugates **1**–**4** and **D1**, **D2** in PBS (excitation at 520 nm).

**Figure 14:**
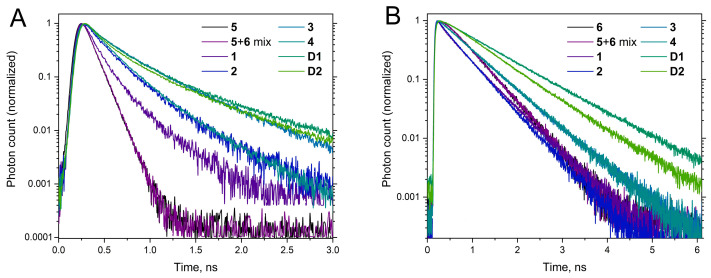
Fluorescence decay kinetics of **1**–**6**, an equimolar **5**+**6** mixture**, D1** and **D2** in PBS; (**A**) sCy3 fluorescence, ex. 510 nm, em. 560 nm; (**B**) sCy5 fluorescence, ex. 640 nm, em. 680 nm.

To further evaluate the dye interactions in conjugates **1**–**4**, fluorescence emission spectra were recorded with excitation only in the sCy3 absorption band (Figure 13). In this case, we observed sensitized sCy5 fluorescence, which indicates energy transfer in all the conjugates. Indeed, the fluorescence decay kinetics were monoexponential for individual dyes **5** and **6** and biexponential for all the conjugates (Figure 14), which is due to the energy transfer between sCy3 and sCy5. As the linker length increases, the energy transfer efficiency decreases significantly. Conjugates **1**–**4**, **D1** and **D2** exhibit a slower fluorescence decay compared to individual dyes. This effect is more pronounced in the case of sCy3, which is more affected by *cis*–*trans* isomerization compared to sCy5 [48]. Among the conjugates, the oligonucleotide-based **D1** and **D2** predictably display the longest fluorescence lifetimes because of their large molecular weight.

The use of the cyanine dye system for quantitative estimation of binding in the case of covalent conjugates encounters a number of difficulties. We have found that the change in the ratio of sCy3/sCy5 0–0 band intensities during conjugate formation depends on the type and length of the linker between the dyes. In this case, the shape of the absorption spectrum changes only for sCy3 but not for sCy5; moreover, sCy5 also showed less sensitivity to the presence of the linker in terms of photophysical properties. Consequently, we can assume that there is little or no change in the molar absorption coefficient of sCy5 in a dye conjugate, and therefore the change in the sCy3/sCy5 0–0 band intensity ratio is primarily due to the change in the molar absorption coefficient of sCy3. Under this assumption, the observed data indicate that an increase in linker length leads to an increase in the molar absorption coefficient of sCy3. We do not consider dimerization, nor the reverse process, as we found no evidence for the presence of aggregated forms of cyanine dyes in our system. It is possible that the molar absorption coefficient also increases for sCy5, but not as significantly due to the lower sensitivity of the structure of the sCy5 molecule to changes at its periphery.

In the case of oligonucleotide linkers, however, the situation is reversed. For the **D2** conjugate, we observe a sCy3/sCy5 band intensity ratio of 0–0 which is practically indistinguishable from that of a simple dye mixture. This suggests that some of the spectral changes in the complete non-oligonucleotide conjugate may be due, in part, to direct interaction of the dyes with each other. Indeed, the linkers we used in low-molecular-weight dye conjugates are flexible, allowing the formation of compact structures where cyanine dyes can be placed quite close to each other, whereas oligonucleotide linkers are rigid enough to prevent such interaction.

The possibility of a direct interaction between the dyes in the low-molecular-weight conjugate is also supported by the results seen in Figure 8 and Figure 9, where the spectral properties of sCy3 and sCy5 differ significantly between **4c**/**4d** and conjugate **4**, in which a molecule of the second dye is added to the opposite end of the linker. In this case, the observed influence of the ionic strength of the solution can be attributed to an increase in the compactness of the conjugate surrounded by counterions; the influence of DMSO can be explained by a direct increase in microviscosity. However, it should be noted that the influence of NaCl and DMSO is more pronounced for sCy5, since optical density in the sCy5 region increases faster than in the sCy3 absorption region, leading to a decrease in the sCy3/sCy5 0–0 band intensity ratio with increasing ionic strength or viscosity of the solution.

In connection with the hypothesis suggesting compaction of the sCy3–sCy5 conjugate, it is necessary to discuss the potential mechanisms of energy transfer from sCy3 and sCy5 observed in such a system (see Figure 13, left). On the one hand, as linker length decreases, the quenching of sCy3 fluorescence increases together with the sensitized fluorescence of sCy5. On the other hand, in conjugate **4**, we cannot calculate the energy transfer efficiency in the FRET model because the fluorescence lifetime of sCy3 does not decrease in the presence of sCy5, but increases (compare **4c** and **4** in Figure 9A). For conjugate **4**, we would expect a significant decrease in the fluorescence lifetime of the energy donor (sCy3) since the length of the linker in the maximally stretched state is comparable to the value of the Förster radius of the sCy3–sCy5 pair. The fluorescence lifetime of sCy3 in the sCy3–sCy5 conjugates (Figure 14A) is primarily determined by the influence of the linker. The increase in energy transfer efficiency with increasing length of the low-molecular-weight linker might be attributed to the fact that a longer linker can provide more conformations for close contact of the two dyes in the folded conjugate **3**.

Overall, the exact reason for the abnormal absorbance ratio in sCy3-sCy5 conjugates remains unclear. We have not found any direct spectroscopic evidence pointing to dye dimer formation, which could explain the aberrations in molar absorption coefficients. In the case of the oligonucleotide-based conjugate **D1**, the dyes are separated by a 12 nm DNA duplex, yet the absorbance ratio is still higher than expected. Only in the case of the extended conjugate **D2** does the ratio become close to that of a mixture of individual dyes. Our findings suggest that caution should be exercised when relying on relative absorbance to determine the molar ratio of chromophores, in particular the sCy3/sCy5 pair.

## 3. Materials and Methods

### 3.1. General Methods

Tetraethylene glycol monopropargyl ether [49], *O*-(2-azidoethyl)triethylene glycol [50], **2** [30], **4a** [30], and **4b** [31] were synthesized as reported previously. Cyanine dye derivatives **S1**–**S5** (Figure S7, Supplementary Materials) were obtained from Lumiprobe LLC (Moscow, Russia). The reactions were monitored by thin-layer chromatography (TLC), carried out on 0.20 mm 60 F254 silica gel plates (Merck, Darmstadt, Germany). The HPLC/MS analysis of low molecular conjugates was carried on an Agilent 1100 HPLC system (Agilent Technologies, Santa Clara, CA, USA) with a diode-array detector and an MSD detector (G1964B) in a gradient of acetonitrile from 5% to 80% for 15 min in 50 mM HFIP-DIPA aqueous buffer (YMC triart C8 column, 250 mm length, 4.6 mm inner diameter). Detection was at 550 nm for derivatives of sulfo-Cy3, or at 650 nm for derivatives containing only sulfo-Cy5.

HPLC of oligonucleotides was carried out on an Agilent 1100 instrument using a Waters xBridge column 4.6 × 250 mm, linear gradient from 5 to 50% MeCN in gradient of triethylammonium acetate from 0.1 M to 0.02 M for 30 min (detection at 260 nm). Oligonucleotides were assembled in an ASM-2000 DNA synthesizer using the phosphoramidite method according to the manufacturer’s recommendations. Protected 2′-deoxyribonucleoside 3′-phosphoramidites, Unylinker-CPG (1000 Å), were purchased from ChemGenes (Wilmington, MA, USA); 5′-alkyne phosphoramidite was from Lumiprobe LLC. Oligonucleotides were treated with 10% diethylamine in MeCN for 10 min, then cleaved from the support and deprotected using AMA—1:1 (*v*/*v*) conc. aq. ammonia and 40% aq. methylamine for 20 min at 65 °C.

### 3.2. Synthesis of Low-Molecular-Weight Conjugates

#### 3.2.1. Conjugate **1**

In a 1.5 mL polypropylene microcentrifuge tube, sCy3 azide **S1** (1.0 mg, 1.4 μmol, 1 equiv.) and sCy5 alkyne **S2** (1.1 mg, 1.4 mmol, 1 equiv.) were dissolved in DMSO (40 μL). An aqueous solution of the CuSO_4_∙THPTA 1:1 complex (99 mM, 14 μL, 1.4 μmol, 1 equiv.) was added, followed by ascorbic acid (284 mM in H_2_O, 50 μL, 14 μmol, 10 equiv.). The reaction mixture was briefly vortexed and agitated on an orbital shaker at 23 °C. After 30 min, EtOAc (1.4 mL) was added to the reaction mixture, the resulting mixture was vortexed and centrifuged, and the supernatant was removed. Addition of EtOAc and centrifugation was performed two more times, resulting in an amorphous dark purple precipitate, which was dried in vacuo. A 0.1 mg portion of the crude product was purified by HPLC, yielding **1** as a purple solid. LCMS (ESI) calcd for C_68_H_81_N_9_O_14_S_4_^2−^ [M − 2H]^2−^ 687.74, found 687.75.

#### 3.2.2. Conjugate **3**

In a 1.5 mL polypropylene microcentrifuge tube, **4a** [30] (Et_3_NH^+^ form, 1.7 mg, 1.5 μmol, 1 equiv.) and sCy5-DBCO, **S3** (1.5 mg, 1.5 mmol, 1 equiv.) were dissolved in DMSO (140 μL). The solution was agitated on an orbital shaker at 23 °C for 16 h, at which point TLC analysis (silica gel, DCM–MeOH–H_2_O–Et_3_N, 85:15:2:1) indicated full conversion of starting material. The crude product was then precipitated in a 2 mL polypropylene microcentrifuge tube by treating 70 μL aliquots of the reaction mixture with 1.8 mL EtOAc followed by centrifugation and supernatant removal. The resulting amorphous dark purple precipitate was washed with EtOAc (3 × 1.8 mL) and dried in vacuo. A 0.1 mg portion of the crude product was purified by HPLC, yielding **3** as a purple solid. LCMS (ESI) calcd for C_96_H_116_N_10_O_20_S_4_^2−^ [M − 2H]^2−^ 928.36, found 928.35.

#### 3.2.3. Conjugate **4**

In a 1.5 mL polypropylene microcentrifuge tube, **4a** (Et_3_NH^+^ form, 2.0 mg, 1.8 μmol, 1 equiv.) and **4b** [30] (Et_3_NH^+^ form, 2.1 mg, 1.8 μmol, 1 equiv.) were dissolved in DMSO (40 μL). An aqueous solution of the CuSO_4_∙THPTA 1:1 complex (99 mM, 18 μL, 1.8 μmol, 1 equiv.) was added, followed by ascorbic acid (284 mM in H_2_O, 64 μL, 18 μmol, 10 equiv.). The reaction mixture was briefly vortexed and agitated on an orbital shaker at 23 °C. After 30 min, TLC analysis (silica gel, DCM–MeOH–H_2_O–Et_3_N, 85:17.5:2:1) indicated full conversion of starting material, and EtOAc (1.4 mL) was added to the reaction mixture. The resulting mixture was vortexed and centrifuged, and the supernatant was removed. Addition of EtOAc and centrifugation was performed two more times, resulting in an amorphous dark purple precipitate, which was dried in vacuo. A 0.1 mg portion of the crude product was purified by HPLC, yielding **4** as a purple solid. LCMS (ESI) calcd for C_91_H_123_N_9_O_25_S_4_^2−^ [M − 2H]^2−^ 934.88, found 935.10.

#### 3.2.4. Compound **4c**

In a 1.5 mL polypropylene microcentrifuge tube, a solution of **4a** (Et_3_NH^+^ form, 1.1 mg, 1.0 μmol, 1 equiv.) in DMSO (29 μL) was mixed with tetraethylene glycol monopropargyl ether [49] (3.3 mg, 14 μmol, 14 equiv.). To the resulting mixture, an aqueous solution of the CuSO_4_∙THPTA 1:1 complex (99 mM, 10 μL, 0.99 μmol, 0.99 equiv.) was added, followed by ascorbic acid (284 mM in H_2_O, 35 μL, 9.9 μmol, 9.9 equiv.). The reaction mixture was briefly vortexed and agitated on an orbital shaker at 23 °C. After one minute, TLC analysis (silica gel, DCM–MeOH–H_2_O–Et_3_N, 85:17.5:2:1) indicated full conversion of **4a**. After 30 more min, EtOAc (1.4 mL) was added to the reaction mixture, the resulting mixture was vortexed and centrifuged, and the supernatant was removed. Addition of EtOAc and centrifugation was performed two more times, resulting in an amorphous dark magenta precipitate, which was dried in vacuo. A 0.1 mg portion of the crude product was purified by HPLC, yielding **4c** as a magenta solid. LCMS (ESI) calcd for C_54_H_78_N_6_O_17_S_2_^2−^ [M − 2H]^2−^ 573.24, found 572.64.

#### 3.2.5. Compound **4d**

In a 1.5 mL polypropylene microcentrifuge tube, a solution of **4b** (Et_3_NH^+^ form, 0.1 mg, 0.086 μmol, 1 equiv.) in DMSO (30 μL) was mixed with *O*-(2-azidoethyl)triethylene glycol [49] (2.5 mg, 11.4 μmol, 130 equiv.). To the resulting mixture, an aqueous solution of the CuSO_4_∙THPTA 1:1 complex (99 mM, 10 μL, 0.99 μmol, 11.5 equiv) was added, followed by ascorbic acid (284 mM in H_2_O, 35 μL, 9.9 μmol, 115 equiv). The reaction mixture was briefly vortexed and agitated on an orbital shaker. After 3 min, TLC analysis (silica gel, DCM–MeOH–H_2_O–Et_3_N, 85:17.5:2:1) indicated full conversion of **4b**. After 10 more min, EtOAc (1.4 mL) was added to the reaction mixture, the resulting mixture was vortexed and centrifuged, and the supernatant was removed. Addition of EtOAc and centrifugation was performed two more times, resulting in an amorphous dark blue precipitate, which was dried in vacuo and purified by HPLC, yielding **4d** as a dark blue solid. LCMS (ESI) calcd for C_56_H_80_N_6_O_17_S_2_^2−^ [M − 2H]^2−^ 586.25, found 585.64.

#### 3.2.6. Compound **4e**

In a round-bottom flask, sCy3 diacid Et_3_NH^+^ salt **S4** (77.7 mg, 0.076 mmol, 1 equiv.) was dissolved in DMF (2 mL), and DIPEA (16 μL, 12 mg, 0.092 mmol, 1.2 equiv.) was added. To the resulting mixture, a solution of TSTU (22 mg, 0.076 mmol, 1 equiv.) in DMF (1 mL) was added dropwise over the course of 15 min. The mixture was stirred at 23 °C for 15 min, after which TLC (silica gel, DCM–MeOH–H_2_O–Et_3_N, 85:17.5:2:1) indicated the formation of mono- and bis-NHS esters. After 20 more min, concentrated aqueous NH_3_ (16 μL, 14.7 M, 0.235 mmol, 3.1 equiv.) was added. The resulting mixture was stirred at 23 °C for 40 min and poured into Et_2_O (60 mL), with an amorphous magenta precipitate forming. The resultant mixture was passed through a pad of Celite, the precipitate was washed with Et_2_O, dried, eluted with MeOH, and concentrated under reduced pressure to give a glassy magenta solid. A 0.3 mg portion of the crude product was purified by HPLC, yielding **4e** as a magenta solid. LCMS (ESI) calcd for C_35_H_44_N_3_O_9_S_2_^−^ [M − H]^−^ 714.25, found 713.64.

#### 3.2.7. Compound **4f**

In a round bottom flask, sCy5 diacid Et_3_NH^+^ salt **S5** (58.3 mg, 0.056 mmol, 1 equiv.) was dissolved in DMF (2 mL), and DIPEA (12 μL, 8.9 mg, 0.069 mmol, 1.2 equiv.) was added. To the resulting mixture, a solution of TSTU (16 mg, 0.056 mmol, 1 equiv.) in DMF (1 mL) was added dropwise over the course of 20 min. The mixture was stirred at 23 °C for 70 min, after which TLC (silica gel, DCM–MeOH–H_2_O–Et_3_N, 85:17.5:2:1) indicated the formation of mono- and bis-NHS esters, and concentrated aqueous NH_3_ (12 μL, 14.7 M, 0.176 mmol, 3.1 equiv.) was added. The resulting mixture was stirred at 23 °C for 2.5 h and poured into Et_2_O (60 mL), with a blue precipitate forming. The resultant mixture was passed through a pad of Celite, the precipitate was washed with Et_2_O, dried, eluted with MeOH, and concentrated under reduced pressure to give a dark blue solid. A 0.3 mg portion of the crude product was purified by HPLC, yielding **4f** as a blue solid. LCMS (ESI) calcd for C_37_H_46_N_3_O_9_S_2_^−^ [M − H]^−^ 740.27, found 739.55.

### 3.3. General Procedure for Oligonucleotide Labeling

Two milliliter tubes with alkyne-modified oligonucleotides (20 nmol) were charged with pre-degassed deionized water (6 μL), 2M TEAA buffer (6 μL), DMSO (25 μL), and azide of dye in DMSO (2 μL of 10 mM solution). The obtained mixture was thoroughly stirred. 10 mM solution of CuSO_4_∙5H_2_O–TBTA premix in 55% aq. DMSO was added (6 μL) to the reaction mixtures. The reactions were started by addition of 10 mM aq. ascorbic acid (15 μL). Tightly closed tubes were kept in darkness for 2 h.

The reaction mixtures were precipitated with 2% LiClO_4_ in acetone (1.5 mL) at −20 °C for 1 h. Centrifugation at 10,000 rcf gave the precipitate, which was rinsed with pure acetone, dried, and dissolved in 50% aq. formamide (100 μL). The products were separated by denaturing 20% polyacrylamide gel electrophoresis. The bands containing products of click reactions were carefully cut with scalpel. The obtained slices were frozen at −20 °C, crushed, and eluted twice with deionized water (500 μL total). Eluates were desalted using NAP-10 gel columns according to the standard protocol.

### 3.4. Oligonucleotide Duplex Formation

Stock 200 μM solutions of purified oligonucleotides in PBS were prepared using absorbance at 260 nm. Molar concentrations were calculated using the standard procedure [51]. The initial sulfo-cyanine labeled oligonucleotides were mixed in PBS buffer in a 1:1 molar ratio to obtain 100 μM final concentrations (sCy3-A1+sCy5-B1 for **D1**, sCy3-A2+sCy5-B2 for **D2**). The resulting mixtures were heated to 95 °C for 5 min and slowly cooled to room temperature. The resulting duplex solutions were diluted with 6× glycerol loading buffer and preparatively separated by native 12% polyacrylamide gel electrophoresis at a constant voltage of 45 V/cm (1 h). The formation of duplexes was confirmed by parallel running of non-hybridized single-stranded labeled oligonucleotides. Pure duplexes containing a pair of covalently-attached sulfo-cyanine dyes were carefully cut from the gel with a scalpel and eluted from the crushed gel slices with PBS.

### 3.5. Absorbance and Fluorescence Spectra

Absorption spectra were recorded using a MayaPro 2000 spectrophotometer (Ocean Optics, Dunedin, FL, USA) and an SLS204 deuterium lamp as a white light source (Thorlabs, Newton, NJ, USA) in PBS.

Fluorescence spectra were recorded on a Varian Cary Eclipse fluorescence spectrometer with excitation wavelength 520 nm, excitation slit 10 nm, and emission slit 10 nm, in a range of fluorescence emission from 535 nm to 850 nm with a rate of 100 nm/min.

### 3.6. Fluorescence Lifetime Measurements

Fluorescence decay kinetics with picosecond time resolution were recorded using a time-correlated single-photon counting mode (TCSPC) based on a SimpleTau-130EM module (Becker & Hickl, Berlin, Germany). For sCy3 excitation, a PLS-510 LED laser (InTop, Saint Petersburg, Russia) was used in picosecond pulse generation mode (25-ps pulses (FWHM) at a repetition rate of 25 MHz) at 510 nm wavelength. For sCy5 excitation, 2nd harmonic (ASG-O-1250-AT) of a Yb femtosecond laser (TEMA-150 modulated by parametric generator TOPOL-1050C, Avesta Project Ltd., Moscow, Russia) was used (150-fs pulses (FWHM) at a repetition rate of 80 MHz) at a wavelength of 640 nm. The sCy3/sCy5 fluorescence was collected using an ML 44 monochromator (Solar Laser Systems, Minsk, Belarus) at 580 and 680 nm, respectively, and the fluorescence decay curves were recorded in single photon counting mode using an HPM-100–07C hybrid detector (Becker & Hickl) with ultra-low background noise. Measurements were carried out in a Qpod 2e thermostatic cuvette holder (Quantum Northwest, Liberty Lake, WA, USA) at 25 °C in a 100 μL quartz cuvette.

## Data Availability

Data are contained within the article and Supplementary Materials.

## Supplementary Materials

The following supporting information can be downloaded at: https://www.mdpi.com/article/10.3390/molecules30010057/s1, Table S1: Oligonucleotide sequences; Figure S1: Scheme of oligonucleotide conjugate synthesis; Figure S2: Native 12% PAGE of duplexes containing Cy3/Cy5 dyes; Figure S3: Absorption spectra of **6** and the equimolar mixture **5** + **6** in PBS; Figure S4: Fluorescence decay kinetics of **5** (A) and **6** (B) measured in individual solutions in mQ water and in equimolar **5** + **6** mixture; Figure S5: Absorbance spectra of dyes **5** (A) and **6** (B) in 10 mM PBS in the presence of various NaCl concentrations; Figure S6: Absorbance spectra of dyes **5** (A) and **6** (B) at constant concentrations in deionized water in the presence of DMSO at various concentrations (0–30% *v*/*v*); Figure S7: Structures of cyanine starting materials; Figure S8: HPLC profiles of low-molecular Cy3/Cy5 conjugates **1**–**4**; Figure S9: HPLC profiles of low-molecular-weight compounds **4a**–**f**. Figure S10: Mass spectra of low-molecular Cy3/Cy5 conjugates **1**–**4**; Figure S11: Mass spectra of low-molecular-weight compounds **4a**–**f**.
