# Supplementary material for: Indocarbocyanine–Indodicarbocyanine (sCy3–sCy5) Absorptive Interactions in Conjugates and DNA Duplexes
Source: Molecules. 2024 Dec 27;30(1):57. doi: 10.3390/molecules30010057 (PMC11721635; doi:10.3390/molecules30010057)
Supplement: Supplementary file 1 [file molecules-30-00057-s001.zip › molecules-3351053-supplementary.pdf]

Supporting Information

Indocarbocyanine–indodicarbocyanine (sCy3–sCy5) absorptive interactions in conjugates and DNA duplexes

Evgeny L. Gulyak, Vladimir A. Brylev, Mikhail Y. Zhitlov, Olga A. Komarova, Alexey V. Ustinov, Ksenia A. Sapozhnikova, Vera A. Alferova, Vladimir A. Korshun and Daniil A. Gvozdev

1. Oligonucleotide conjugates

Table S1. Oligonucleotide sequences

Name	Sequence, 5′→3′	Length
A1	[Alkyne]GGTCGCTTATCTGCACTCGGA	21
B1	[Alkyne]TCCGAGTGCAGATAAGCGACC	21
A2	[Alkyne]TTTTTTTTGGTCGCTTATCTGCACTCGGA	29
B2	[Alkyne]TTTTTTTTTCCGAGTGCAGATAAGCGACC	29

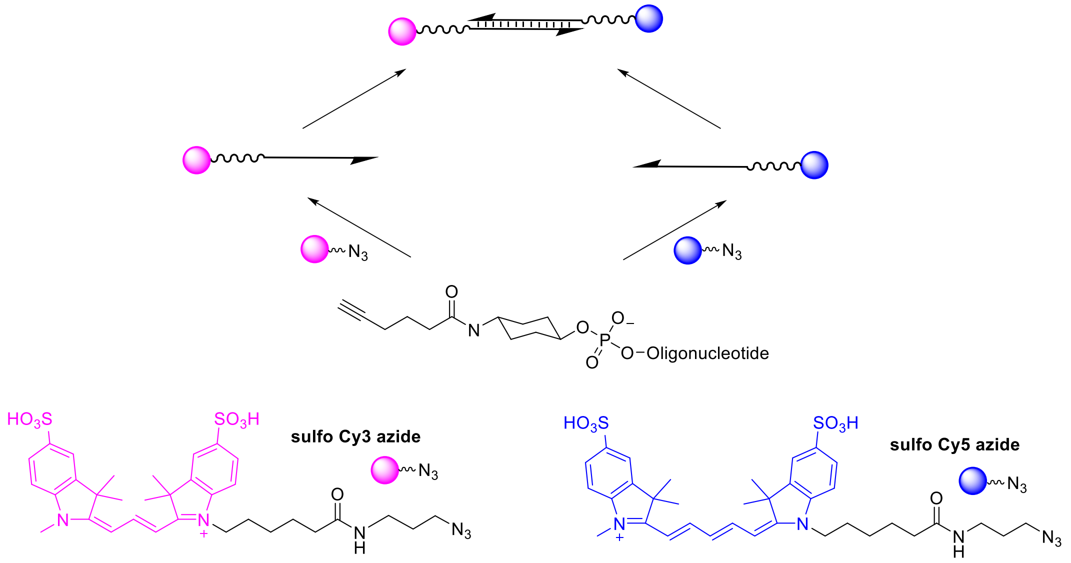

Figure S1. Scheme of oligonucleotide conjugate synthesis.

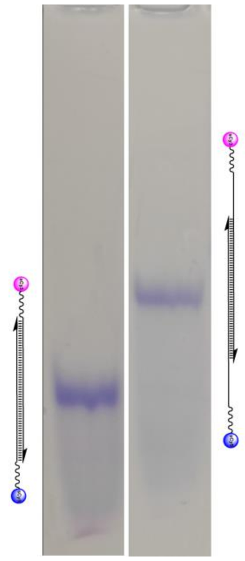

Figure S2. Native 12% PAGE of duplexes containing Cy3/Cy5 dyes.

2. Spectral and photophysical properties of Cy3–Cy5 conjugates

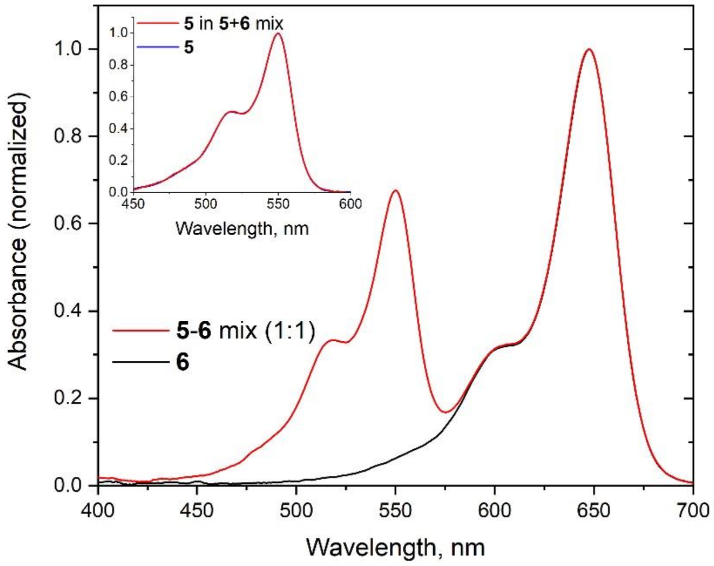

Figure S3. Absorption spectra of **6** and the equimolar mixture **5**+**6** in PBS. Inset: absorption spectra of **5** in mQ water and **5** calculated by subtracting the spectrum of **6** from the **5**+**6** mixture spectrum.

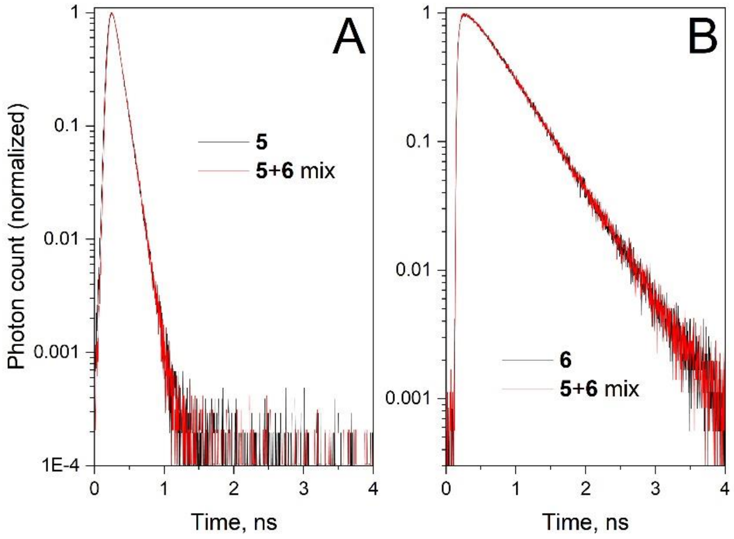

Figure S4. Fluorescence decay kinetics of **5** (A) and **6** (B) measured in individual solutions in mQ water and in equimolar **5**+**6** mixture.

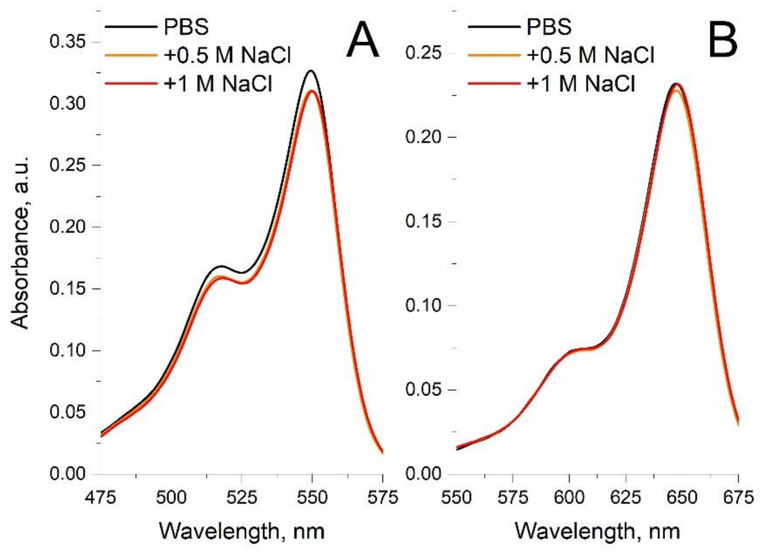

Figure S5. Absorbance spectra of dyes **5** (A) and **6** (B) in 10 mM PBS in the presence of various NaCl concentrations.

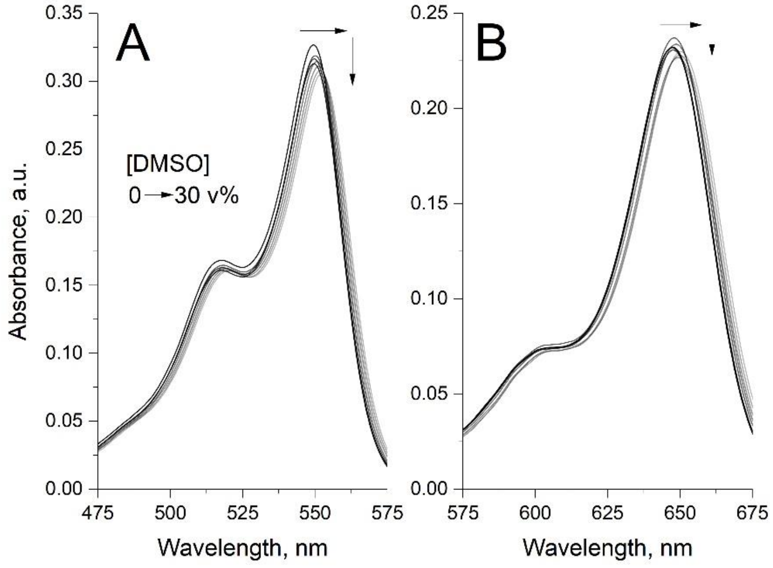

Figure S6. Absorbance spectra of dyes **5** (A) and **6** (B) at constant concentrations in deionized water in the presence of DMSO at different concentrations (0–30% *v*/*v*). Arrows show changes in the spectral properties of the dyes with increasing DMSO concentration.

3. Synthesis and characterization of low-molecular-weight conjugates

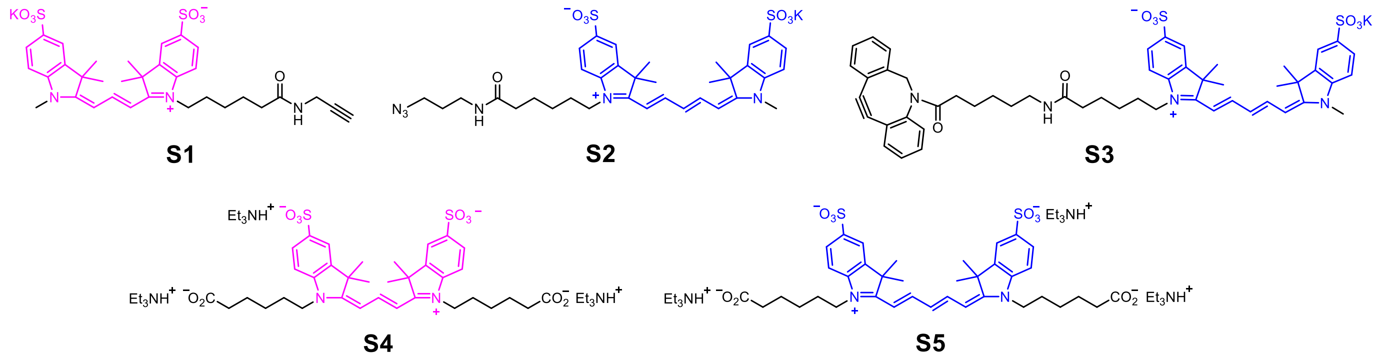

Figure S7. Structures of cyanine starting materials.

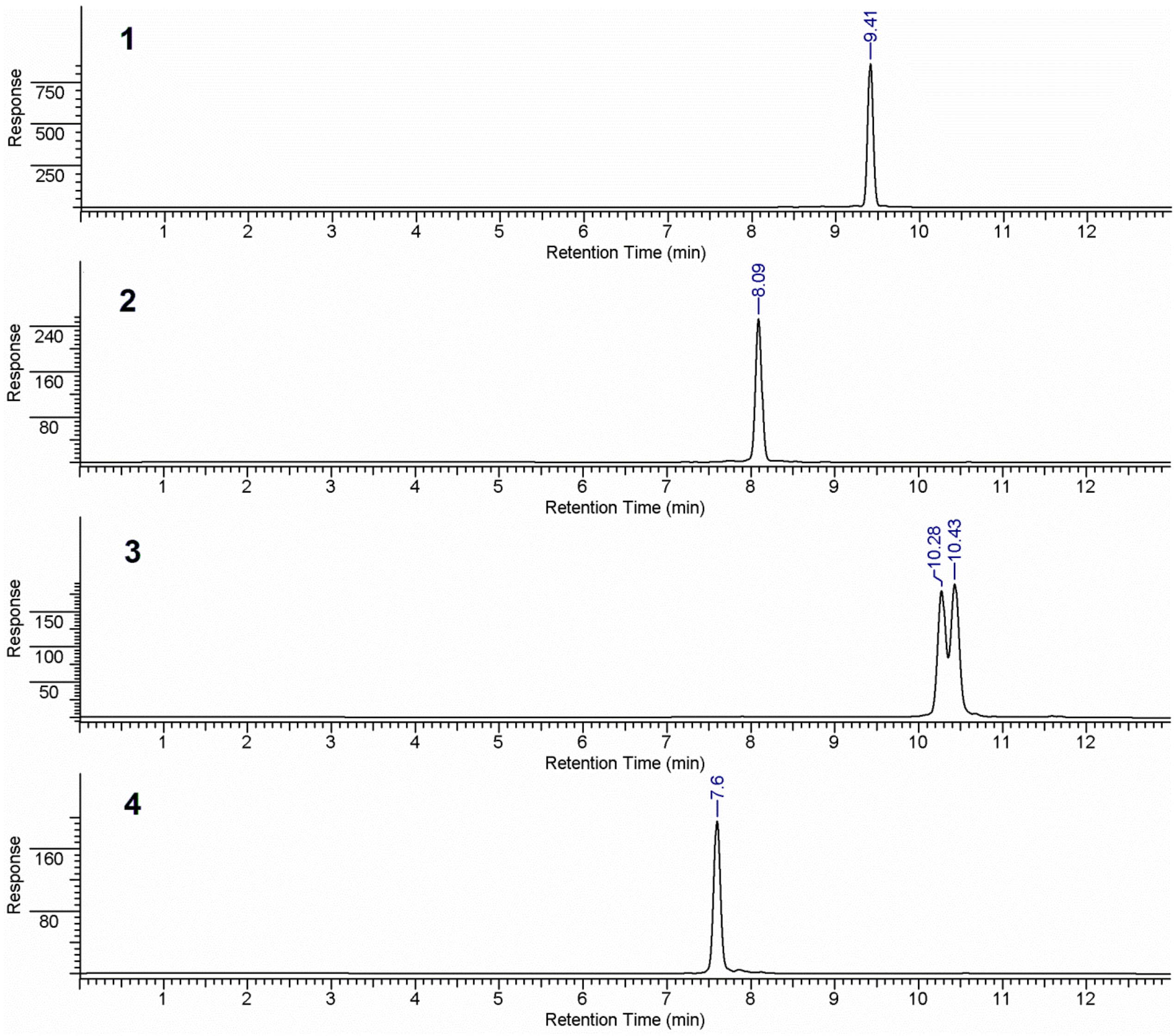

Figure S8. HPLC profiles of low-molecular-weight Cy3–Cy5 conjugates **1**–**4**.

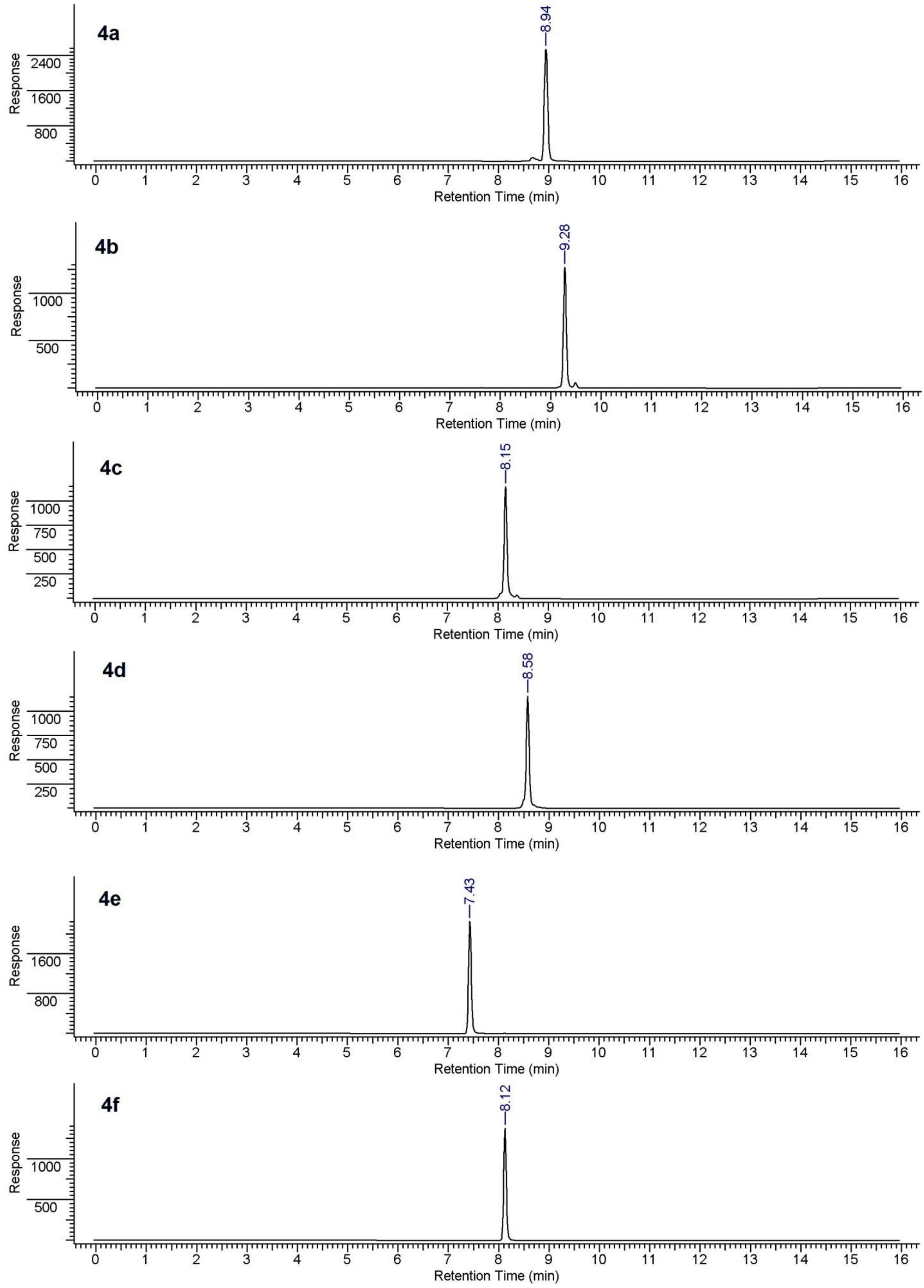

Figure S9. HPLC profiles of low-molecular-weight compounds **4a–f**.

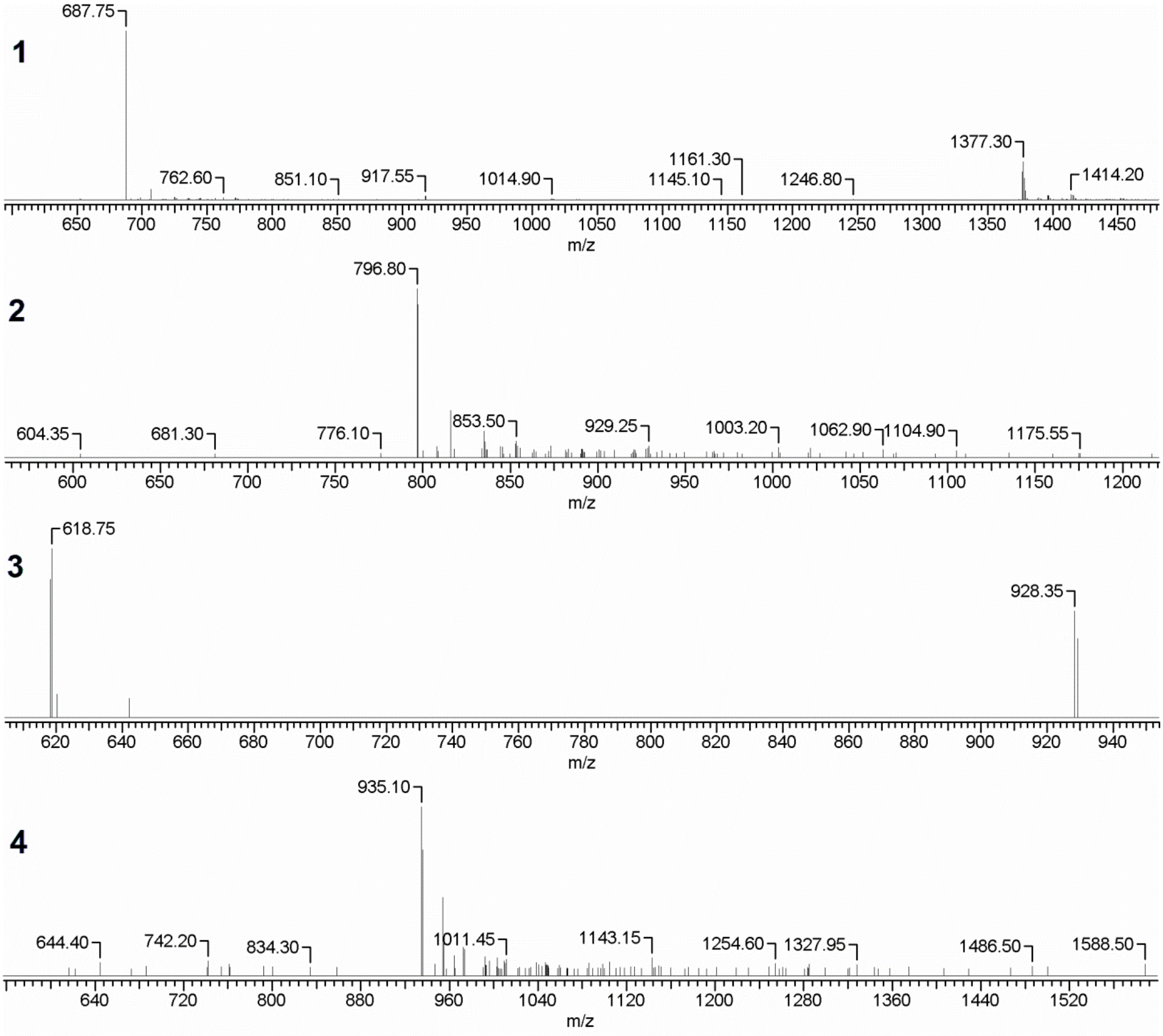

Figure S10. Mass spectra of low-molecular-weight Cy3–Cy5 conjugates **1–4**.

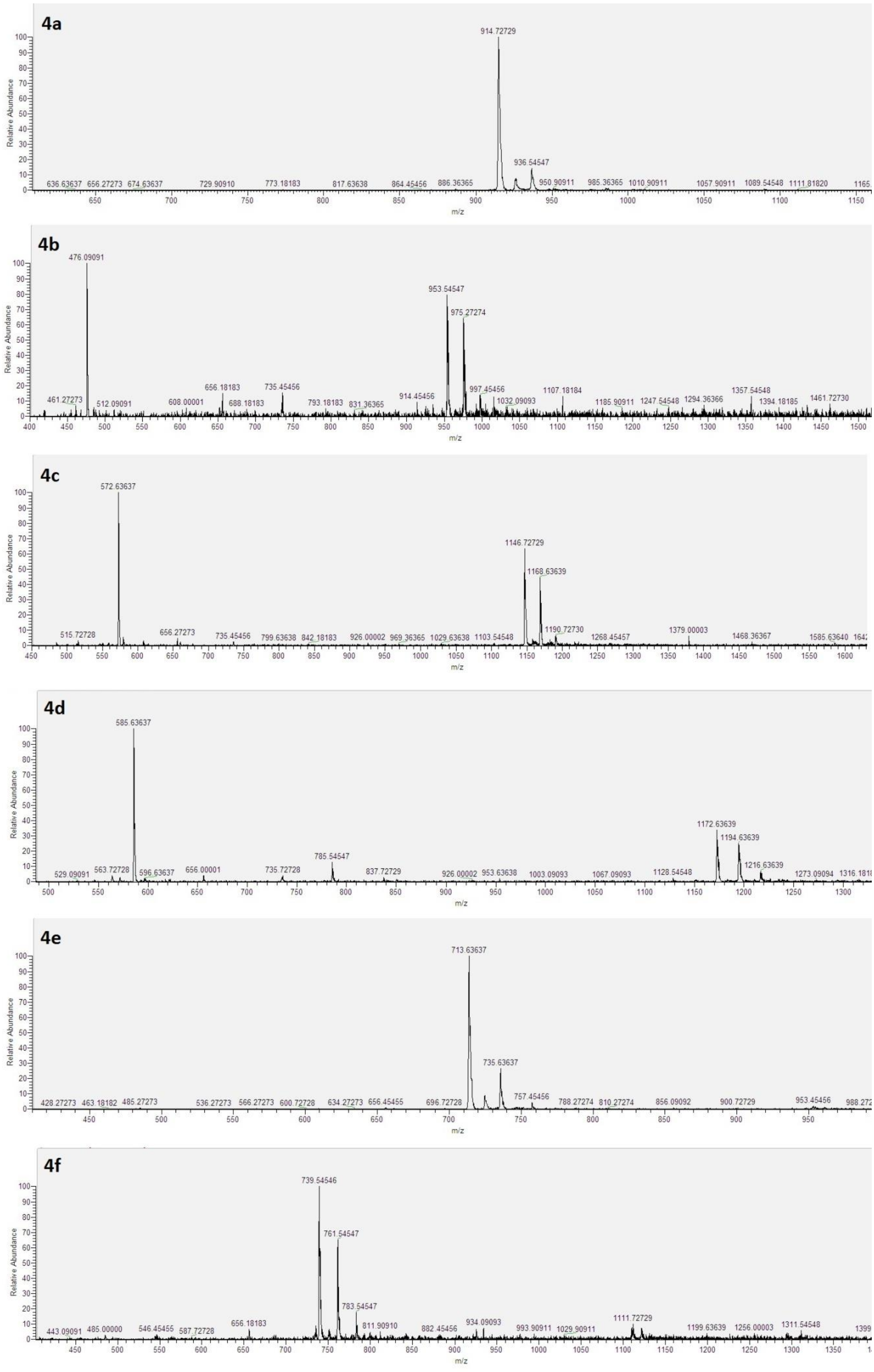

Figure S11. Mass spectra of low-molecular-weight compounds **4a–f**.
